# SARS-CoV-2 in patients with cancer: possible role of mimicry of human molecules by viral proteins and the resulting anti-cancer immunity

**DOI:** 10.1007/s12192-021-01211-7

**Published:** 2021-05-11

**Authors:** Stefano Burgio, Everly Conway de Macario, Alberto JL Macario, Francesco Cappello

**Affiliations:** 1 Department of Biomedicine, Neurosciences and Advanced Diagnostics (BIND), Institute of Human Anatomy and Histology, University of Palermo, 90141 Palermo, Italy; 2 Department of Microbiology and Immunology, School of Medicine, University of Maryland at Baltimore-Institute of Marine and Environmental Technology (IMET), Baltimore, MD 21202 USA; 3 Euro-Mediterranean Institute of Science and Technology (IEMEST), 90139 Palermo, Italy

**Keywords:** COVID-19, SARS-CoV-2, Cancer, Molecular mimicry, Shared epitopes, Immunological cross-reaction

## Abstract

A few reports suggest that molecular mimicry can have a role in determining the more severe and deadly forms of COVID-19, inducing endothelial damage, disseminated intravascular coagulation, and multiorgan failure. Heat shock proteins/molecular chaperones can be involved in these molecular mimicry phenomena. However, tumor cells can display on their surface heat shock proteins/molecular chaperones that are mimicked by SARS-CoV-2 molecules (including the Spike protein), similarly to what happens in other bacterial or viral infections. Since molecular mimicry between SARS-CoV-2 and tumoral proteins can elicit an immune reaction in which antibodies or cytotoxic cells produced against the virus cross-react with the tumor cells, we want to prompt clinical studies to evaluate the impact of SARS-CoV-2 infection on prognosis and follow up of various forms of tumors. These topics, including a brief historical overview, are discussed in this paper.

## A historical background

The pandemic caused by the severe acute respiratory syndrome coronavirus 2 (SARS-CoV-2), which causes the coronavirus disease 2019 (COVID-19), has resulted in over 2.53 million deaths as of March 1, 2021, all around the world (Dong et al. 2020). Despite the large number of publications that have appeared over the last few months, detailed information on pathogenic mechanisms is still scarce (Cappello et al. 2020; Carvalho 2020; Marietta et al. 2020). Noteworthy is the near absence of data or commentaries about the impact of SARS-CoV-2 infection on tumors in those cancer patients that have recovered from the former. Our purpose here is to briefly recapitulate a specific aspect of this issue, which might have implications in the management of cancer patients.

The immune system is involved in tumoral diseases, but the how is under scrutiny, even now after many years of work in various laboratories on the role of immunity in cancer onset, progression, and treatment (Gonzalez et al. 2018). The immune system is often ineffective against tumors because of inadequately targeted responses towards the tumor mass, or because of a reprogramming of immune cells that become pro- rather than against cancer, or because of molecular escape mechanisms with which cancer cells hide antigenic epitopes that would otherwise elicit anti-tumor immunity, or a combination of them (Chen et al. 2019; Gonzalez et al. 2018; Messerschmidt et al. 2016). In any case, the overall result in many patients is that the immune system is unable to stop cancer growth and dissemination.

This has been a stumbling block to treat cancers with strategies based on stimulating the immune system, i.e., immunotherapy, to produce antibodies and cells that efficaciously kill the tumor cells. Early studies proposed the use of infectious agents as potential therapeutic tools for stimulating the immune system in the hope that a reaction will occur not only against the immunogen but would also somehow affect the tumor (McCarthy 2006). One of the pioneers in this field was William Coley, an orthopedic surgeon who was considered by some as the father of immunotherapy. In 1891, he experimented with forms of treatment alternatives to surgery for inoperable sarcomas (Coley 1991; McCarthy 2006). Dr. Coley injected patients in situ with *Streptococcus* cultures, first live and later heat inactivated. In most cases, the patients suffered a severe fever and some tumors regressed. However, “Coley’s toxin” was severely criticized by the scientific community for various reasons: (i) patient follow-up was inadequate; (ii) there were too many variants, thirteen in all, of Coley’s toxin producing variable results; and (iii) the methods of administration were not standardized and varied from intravenous administration to the in situ injection of the compound (McCarthy 2006). Consequently, Coley’s findings were set aside for several years, and other treatment strategies were given preference such as radio- and chemotherapies. However, Coley’s work may have contributed to set the foundations of modern immunotherapy (Decker et al. 2017).

More recently, greater attention has been given to the importance of the tumor microenvironment and the immunological dynamics in it, and the possible involvement of viral infections in tumor progression has been reconsidered (Newman et al. 2020). For instance, intratumoral but not intramuscular injections of flu vaccine without adjuvant can change non-responsive tumors that are resistant to immune infiltration and traditional therapies (in particular to checkpoint blockade compounds) and called “cold tumors,” into responsive and susceptible tumors, called “hot tumors,” through a CD8 + T cell-mediated process (Newman et al. 2020). In addition, the intra-tumor injection of the vaccine conferred immunological protection of the patient against viral infection of the lungs (Newman et al. 2020). These findings support the idea that viral and bacterial pathogens may in some cases enhance the immune response to also encompass a tumor.

In this regard, injecting active influenza virus into the lung of a mouse with an implanted human melanoma reduced tumor growth (Newman et al. 2020). In contrast, the injection of active subcutaneous influenza virus did not reduce the growth of the same tumor. The explanation of these diverse results, depending on the site of injection of the virus, may be found in the presence of natural receptors for the virus in the lungs and their absence in the skin. The lung contains sialic acid, a receptor for the influenza virus, whereas the skin does not. A productive infection occurring when the virus finds its natural targets induces an immune response that may also be directed to the tumor (Newman et al. 2020).

These mechanisms of pathogen-host interaction may not always be in favor of the host but may trigger an autoimmune response through molecular mimicry (Cappello et al. 2009). This may be illustrated by *Chlamydia trachomatis* infections that are accompanied by autoimmune phenomena against human molecules, e.g., the chaperone heat shock protein 60 (Hsp60), that share antigenic epitopes with the microbial orthologue (Cappello et al. 2009). This cross-reactivity and its pathological consequences in tissues are amplified by the widespread distribution of Hsp60 in the body both intra- and extracellularly. Similar examples of molecular mimicry are not uncommon in bacterial and viral infections, which explains why many of these infections are accompanied or followed by autoimmune conditions from mild to severe, including generalized thrombosis with multiorgan failure.

## COVID-19 and molecular mimicry in cancer

There are few retrospective studies on the course of COVID-19 pathology in cancer patients, and the existing reports focus on the average survival data in patients with co-morbidities (Carvalho 2020; Kuderer et al. 2020; Lee et al. 2020; Robilotti et al. 2020). However, correlation of antiviral responses with tumor progression or lack thereof might reveal effects against the tumor.

Molecular mimicry may play a role in the pathogenic mechanisms associated with SARS-CoV-2 virus infection (Angileri et al. 2020; Cappello 2020a; Cappello et al. 2020; Cappello 2020b; Hightower and Santoro 2020; Kasperkiewicz 2021; Lucchese and Flöel 2020; Marino Gammazza et al. 2020). Molecular mimicry of human molecules by viral molecules would elicit antibodies (or cytotoxic cells) against the virus that are cross-reactive with the human molecules, and this, in turn, could lead to autoimmune reactions on the vascular endothelium and generalized thrombosis. Furthermore, if the human cross-reactive molecules are present on the surface of tumor cells exposed to the immune system, they could be the focus of autoimmune reactions that might be deleterious for the tumor. Examples of human molecules that share epitopes with microbial counterparts are various types of heat shock proteins/molecular chaperones (Hsp/MolChaps) that normally reside predominantly intracellularly but can migrate toward the cell membrane under conditions of stress like those under which tumor cells must survive. Therefore, it is not surprising that chaperones such as Hsp60 and Hsp70 are displayed on the surface of cancer cells and can be reached by antibodies and immune cells. We have established that SARS-CoV-2 molecules share immunogenic/antigenic epitopes with various molecular chaperones, indicating the possibility of autoimmunity (Marino Gammazza et al. 2020).

## Further considerations and first evidence

**Fig. 1:**
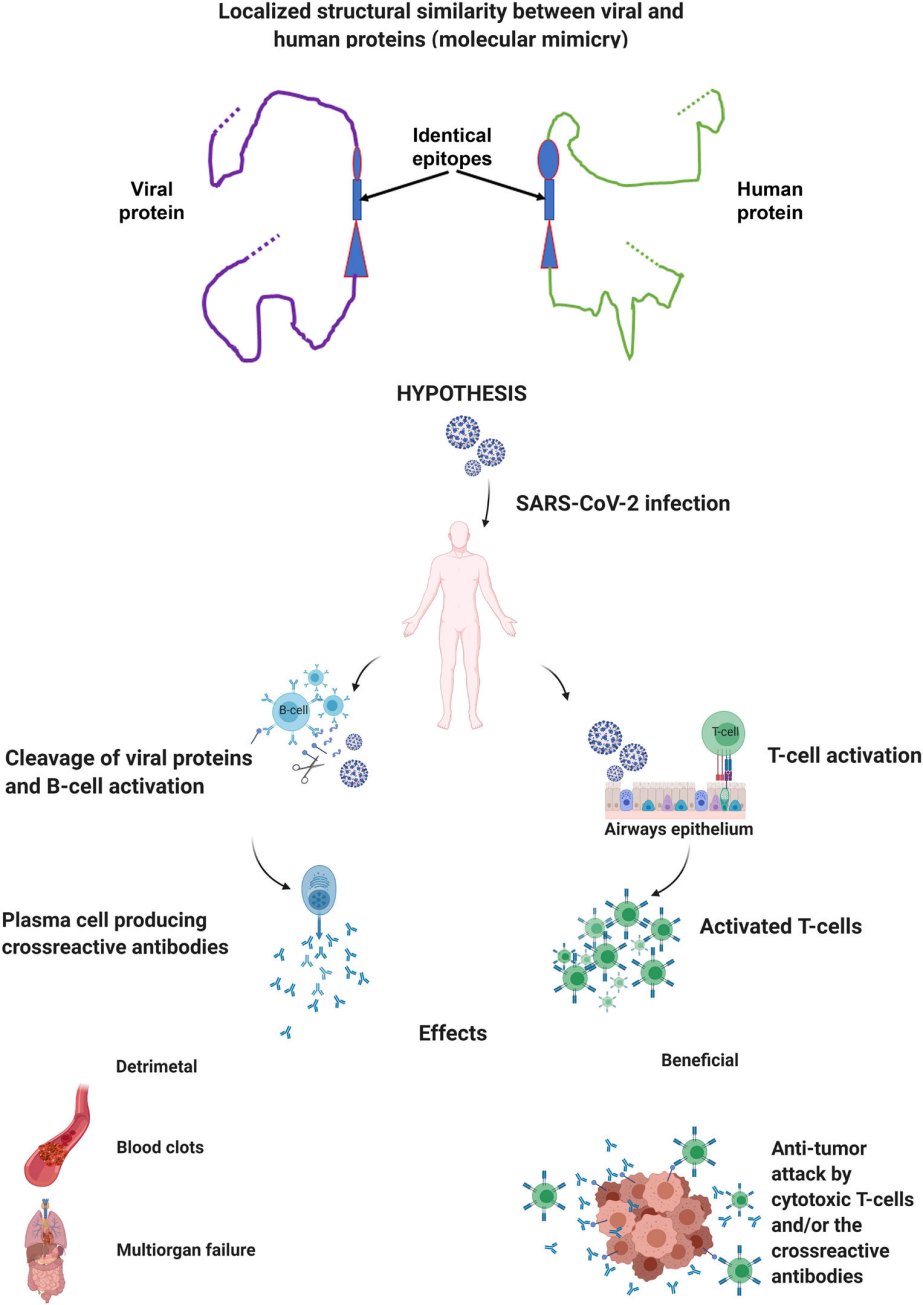
Two proteins, one from the SARS-CoV-2 virus and the other human, may differ in overall size and shape but can share similar antigenic determinants with identical epitopes. Here, the shared identical epitope is represented by a rectangle sitting in between other epitopes that are similar but not identical in the two proteins. An epitope may be formed by a continuous stretch of five or more amino acids in the linear amino acid sequence of the protein as schematized here or by the amino acids being close together in a folded tridimensional protein in such a way that they are recognized as an antigenic epitope. Shared epitopes have the potential of eliciting cross-reactive antibodies and/or killer cells. If the human protein is part of a cancer cell and is exposed to the immune system, antibodies and/or killer cells elicited by the viral protein may react with the tumor cell and cause its lysis. The mechanisms involved would be the same as those operating in anti-cancer immunity elicited by cancer cell antigens in the absence of the virus, but the anti-cancer immune response would be stronger (created with BioRender.com)

SARS-CoV-2 displays considerable immunogenic potential and elicits autoimmunity in the host, most likely attributable to molecular mimicry (Angileri et al. 2020; Cappello 2020a; Cappello et al. 2020; Marino Gammazza et al. 2020). Viral proteins are recognized by the host’s immune system resulting in the production of antiviral antibodies and cytotoxic cells. Since there is considerable sharing of antigenic epitopes between viral and human molecules, the antibodies and cytotoxic cells react not only against the virus but also against the host’s structures mimicked by the viral antigens that occur, for example, on the endothelial cells of the vascular and olfactory systems. These would be the pathogenic mechanism underpinning generalized thrombosis and anosmia, both typical features of many cases of COVID-19 (Fig. 1).

Molecular chaperones are among the molecules that share antigenic/immunogenic epitopes with viral proteins and, therefore, are likely to be the center of autoimmune reactions in COVID-19 patients (Angileri et al. 2020; Hall 2021; Kasperkiewicz 2021; Marino Gammazza et al. 2020). Although molecular chaperones are classically considered intracellular molecules, they can also reside on the cell membrane and extracellularly, including in body fluids in circulation. Noteworthy is the increase of chaperones like Hsp60, Hsp70, and Hsp90 in cancer cells, which display them on their surface (Cappello et al. 2020; Marino Gammazza et al. 2020) and, thus, become available for contact with antibodies and cytotoxic cells elicited by SARS-CoV-2 against antigens shared by viral molecules with human Hsp/MolChaps.

There is considerable evidence in the literature showing that viral and bacterial infections can enhance the immune response against tumors (Cappello et al. 2009; Newman et al. 2020). What happens if the cross-reactive structures are located on the surface of the tumor cells? A recent report describes a 61-year-old patient with progressive lymphadenopathy due to an Epstein–Barr virus (EBV)-positive classical Hodgkin lymphoma that shortly after the diagnosis of the lymphoma was hospitalized due to a SARS-CoV-2 infection and after 4 months from the infection the palpable lymphadenopathy was reduced, and the control PET (positron emission tomography) scan showed a general remission of the tumor pathology, possibly attributable to SARS-CoV-2 infection (Challenor and Tucker 2021).

Along these lines, it appears worthwhile to follow up COVID-19 patients with cancer to monitor tumor progression and determine whether the tumor disease is diminished when there is autoimmunity elicited by the virus. Nascent/subclinical neoplasms might be eliminated during COVID-19 by this mechanism of autoimmunity without being noticed by the patient or physician.

## Conclusions

The currently available data pertaining to oncological patients infected with SARS-CoV-2 are limited to assessing the mortality index in association with different co-morbidities (Kuderer et al. 2020; Lee et al. 2020). Hopefully, new guidelines will be formulated soon for the treatment and management of tumor pathologies in case of COVID-19 co-morbidity.

The classic anticancer therapies are poorly tolerated in COVID-19 patients (Lee et al. 2020). SARS-CoV-2 infection affects much more than the lungs and is a systemic disease, including disseminated thrombosis. All or some of these systemic lesions could be the result of autoimmune reactions mediated by antibodies and cytotoxic cells elicited by viral antigens that cross-react with human structures (Fig. 1).

Similar situations of molecular mimicry have been described involving Hsp/MolChaps and other molecules. Chronic infection by *C. trachomatis*, silent or causing overt disease, can be accompanied by autoimmune manifestations caused by cross-reactive antibodies elicited by the microbial Hsp/MolChaps that cross-react with the host’s counterparts, which although predominantly intracellular can exit the cell or migrate to the cell membrane and become accessible to the immune system (Cappello et al. 2009; Cappello 2020a, b ; Marino Gammazza et al. 2020). The same phenomenon of Hsp/MolChap migration to the cell surface occurs in cancer cells, possibly triggered by stressors such reactive oxygen species (ROS) to which they are exposed (Aggarwal et al. 2019; Dai et al. 2012). Thus, human Hsp/MolChaps or other molecules sharing epitopes with viral antigens become exposed to the immune system on the surface of cancer cells, which may bring about cell death if cytotoxic antibodies or cells made against the virus reach them (Newman et al. 2020).

If indeed a response against cancer cells is elicited by epitopes on viral proteins, the mechanisms involved should be the same as those operating in patients without viral infection when the organism reacts against cancer cell antigens. Other possibilities to consider are, for example, non-specific stimulation of the immune system by viral antigens, but this does not apply here because we are focusing only on cross-reactive antigens shared by viral and cancer molecules. Therefore, what we postulate here is quite different from what happens when the Bacillus Calmette–Guérin (BCG) vaccine induces anti-cancer protection and does not involve innate training or trained immunity (Alhunaidi and Zlotta 2019; Aydillo et al. 2020; Fuge et al. 2015; Guallar-Garrido and Julián 2020).

Within this framework of ideas, we are proposing that it is possible that at least in a subset of COVID-19 patients with cancer, progression of the latter is slowed down, or even interrupted if the tumor is at the initial stages. Ex vivo and in vitro studies should help in testing this hypothesis and unveil molecular details that might be critical indicators for developing novel strategies for managing tumors. Follow-up of COVID-19 patients with cancer by measuring in blood autoantibodies against, for example, human chaperone molecules and correlating the titers with tumor progression (or regression), should be the first step to test the proposed hypothesis. Experimentally, the cytotoxic/cytolytic effect of plasma or serum from patients with autoantibodies could be tested in vitro, against tumor cells or 3D tumoroids.
